# Draft Genome Sequence of the Halophilic and Highly Halotolerant *Gammaproteobacteria* Strain MFB021

**DOI:** 10.1128/genomeA.01156-14

**Published:** 2014-11-20

**Authors:** Toms C. Joseph, Anju Baby, Dinesh Reghunathan, Aswathy Mary Varghese, V. Murugadas, K. V. Lalitha

**Affiliations:** Microbiology, Fermentation and Biotechnology Division, Central Institute of Fisheries Technology, Cochin, Kerala, India

## Abstract

We report the 4.25-Mbp first draft sequence of *Gammaproteobacteria* strain MFB021, a moderate halophile isolated from petroleum-contaminated soil in Cochin, India. The genome of the strain MFB021 was sequenced to understand the mechanism of hydrocarbon degradation and the halophilicity of the bacterium.

## GENOME ANNOUNCEMENT

The class *Gammaproteobacteria* constitutes a very large and diverse group of bacteria that exhibits enormous variety in terms of their phenotype and metabolic capabilities (1, 2). Strain MFB021, identified as belonging to *Gammaproteobacteria*, was isolated from a soil sample contaminated with petroleum and other hydrocarbons in Cochin, India. Bushnell Haas (BH) broth medium supplemented with diesel oil was used for the isolation of strain MFB021. The organism is able to degrade petroleum products, can grow in up to 25% salinity, and can tolerate salinity levels of up to 30%. The genome of the *Gammaproteobacteria* strain MFB021 was sequenced to gain understanding into the mechanisms involved in osmotolerance and petroleum degradation capability.

Sequencing was performed on the Illumina MiSeq platform with a 2 × 250 paired-end run; 522,255 paired sequences were generated, for a total of >193 Mb and a mean length of 185 bases per read. The reads were analyzed and quality checked using FastQC (3). *De novo* assembly was performed using ABySS version 1.3.7 (4). We used SSPACE version 2.0 (5) to extend and merge the resulting scaffolds based on read-pair information, as well as short overlaps to reduce the number of scaffolds, which resulted in 136 contigs and an average coverage of 43×, for a total of 4,436,200 bp. Genome annotation was automatically performed on the RAST server (6), Glimmer 3 (7, 8), GeneMark (9), the KEGG database (10), tRNAscan-SE (11), RNAmmer (12), and Signal P4.1 (13), using Glimmer for base calling, obtaining 3,324 protein-coding genes.

Among the coding sequences (CDSs), 1,332 are not in a subsystem, whereas 1,992 CDSs (1,898 nonhypothetical and 94 hypothetical) are in subsystems. RAST annotation also predicted the involvement of 162 genes in stress responses, including 30 genes involved in osmotic stress (2 in osmoregulation; 21 in choline, betaine uptake, and betaine biosynthesis; 3 in the synthesis of osmoregulated periplasmic glucans; and 4 in ectoine biosynthesis and regulation), 75 in oxidative stress (12 in glutathione:nonredox reactions, 6 in redox-dependent regulation, 6 in the glutathione:redox cycle, 6 in glutaredoxin, 24 in oxidative stress, and 2 in glutaredoxins), 8 in protection from reactive oxygen species (ROS), 3 in cold shock, 15 in heat shock, and 29 in detoxification. Also, 44 genes are involved in the metabolism of aromatic compounds (3 in salicylate ester degradation, 4 in benzoate degradation, 4 in the chloroaromatic degradation pathway, 11 in the catechol branch of the beta-ketoadipate pathway, 5 in salicylate and gentisate catabolism, and 13 in the protocatechuate branch of the beta-ketoadipate pathway). The organism also has 6 genes involved in the synthesis of the plant hormone auxin.

This data set provides insight into the features of this bacteria that may contribute to petroleum utilizing ability and its survival and growth under halophilic conditions.

### Nucleotide sequence accession numbers.

This whole-genome shotgun project has been deposited at DDBJ/EMBL/GenBank under the accession no. JNVT00000000. The version described in this paper is version JNVT01000000.

## References

[1] Woese CR Weisburg WG Hahn CM Paster BJ Zablen LB Lewis BJ Macke TJ Ludwig W Stackebrandt E 1985 The phylogeny of purple bacteria: the gamma subdivision. Syst. Appl. Microbiol. 6:25–33. 10.1016/S0723-2020(85)80007-2. 11541974

[2] Kersters K Devos P Gillis M Swings J Vandamme P Stackebrandt E 2006 Introduction to the Proteobacteria. *In* Dworkin M Falkow S Rosenberg E Schleifer KH Stackebrandt E (ed), The Prokaryotes: A handbook on the biology of Bacteria. Springer Verlag, New York, NY.

[3] Andrews S 2010 FASTQC package. Available online at: http://www.bioinformatics.babraham.ac.uk/projects/fastqc/.

[4] Simpson JT Wong K Jackman SD Schein JE Jones SJ Birol I 2009 ABySS: A parallel assembler for short read sequence data. Genome Res. 19:1117–1123. 10.1101/gr.089532.108. 19251739 PMC2694472

[5] Boetzer M Henkel CV Jansen HJ Butler D Pirovano W 2011 Scaffolding preassembled contigs using SSPACE. Bioinformatics 27:578–579. 10.1093/bioinformatics/btq683. 21149342

[6] Aziz RK Bartels D Best AA DeJongh M Disz T Edwards RA Formsma K Gerdes S Glass EM Kubal M Meyer F Olsen GJ Olson R Osterman AL Overbeek RA McNeil LK Paarmann D Paczian T Parrello B Pusch GD Reich C Stevens R Vassieva O Vonstein V Wilke A Zagnitko O 2008 The RAST server: rapid annotations using subsystems technology. BMC Genomics 9:75. 10.1186/1471-2164-9-75. 18261238 PMC2265698

[7] Delcher AL Bratke KA Powers EC Salzberg SL 2007 Identifying bacterial genes and endosymbiont DNA with glimmer. Bioinformatics 23:673–679. 10.1093/bioinformatics/btm009. 17237039 PMC2387122

[8] Salzberg SL Delcher AL Kasif S White O 1998 Microbial gene identificationusing interpolated Markov models. Nucleic Acids Res. 26:544–548. 10.1093/nar/26.2.544. 9421513 PMC147303

[9] Besemer J Lomsadze A Borodovsky M 2001 GeneMarkS: a selftraining method for prediction of gene starts in microbial genomes. Implications for finding sequence motifs in regulatory regions. Nucleic Acids Res. 29:2607–2618. 10.1093/nar/29.12.2607. 11410670 PMC55746

[10] Lukashin AV Borodovsky M 1998 GeneMark.hmm: new solutions forgene finding. Nucleic Acids Res. 26:1107–1115. 10.1093/nar/26.4.1107. 9461475 PMC147337

[11] Kanehisa M Goto S Kawashima S Okuno Y Hattori M 2004 The KEGG resource for deciphering the genome. Nucleic Acids Res. 32:277–280. 10.1093/nar/gkh063. PMC308797 14681412

[12] Lowe TM Eddy SR 1997 tRNAscan-SE: a program for improved detection of transfer RNA genes in genomic sequence. Nucleic Acids Res. 25:955–964. 10.1093/nar/25.5.0955. 9023104 PMC146525

[13] Lagesen K Hallin P Rødland EA Staerfeldt HH Rognes T Ussery DW 2007 RNAmmer: consistent and rapid annotation of ribosomal RNA genes. Nucleic Acids Res. 35:3100–3108. 10.1093/nar/gkm160. 17452365 PMC1888812

